# The Prognostic Utility of Pathophysiologically Distinct Biomarkers for Renal Outcomes in Sepsis: A Prospective ICU Cohort Study †

**DOI:** 10.3390/jcm14155370

**Published:** 2025-07-30

**Authors:** Mert Canbaz, Günseli Orhun, Özlem Polat, İlkay Anaklı, Abdurrahman Fatih Aydın, Serhat Kılınç, Perihan Ergin Özcan, Figen Esen

**Affiliations:** 1 Department of Anesthesiology and Reanimation, Istanbul Faculty of Medicine, Istanbul University, Millet Cd. Cerrahi Monoblok Giriş Kat., 34093 Istanbul, Turkey; gunseli_orhun@hotmail.com (G.O.); ozlemp@yahoo.com.tr (Ö.P.); ilkayyenicelioglu@gmail.com (İ.A.); persude@gmail.com (P.E.Ö.); esenf@istanbul.edu.tr (F.E.); 2 Department of Medical Biochemistry, Istanbul Faculty of Medicine, Istanbul University, 34093 Istanbul, Turkey; mdfaydin@gmail.com (A.F.A.); kilincsrht@gmail.com (S.K.)

**Keywords:** sepsis-associated acute kidney injury, renal replacement therapy, syndecan-1, NGAL, presepsin, proenkephalin

## Abstract

**Background and Aims:** Sepsis-associated acute kidney injury (S-AKI) is common and is associated with poor outcomes. This prospective observational study aimed to assess the predictive value of four novel biomarkers—syndecan-1 (SDC1), neutrophil gelatinase-associated lipocalin (NGAL), proenkephalin (PENK), and presepsin (PSPN)—for renal outcomes and mortality in septic ICU patients. **Methods:** Serum biomarker levels were measured in serum samples collected at the time of sepsis diagnosis on the basis of the Sepsis-3 criteria. Acute kidney injury (AKI) was defined according to the Kidney Disease: Improving Global Outcomes (KDIGO) guidelines, and patients were grouped by the presence of AKI, renal replacement therapy requirement (RRT), and intensive care unit (ICU) survival. Demographic, clinical, laboratory, and severity score data were compared between groups to evaluate the predictive performance of biomarkers and clinical parameters. **Results:** Of the 140 septic patients included, 55.0% developed AKI, 17.2% required RRT, and the ICU mortality rate was 50.0%. SDC1 was independently associated with both AKI (OR: 1.201; *p* = 0.024) and RRT initiation (OR: 1.260; *p* = 0.004). It also demonstrated the highest predictive performance for RRT (AUC: 0.715; *p* = 0.001) and a significant AUC for AKI evaluation (AUC: 0.659; *p* = 0.002). NGAL levels were significantly elevated in patients with AKI and higher SOFA scores but were not independently predictive. PENK and PSPN were not significantly associated with any renal outcome or mortality. The combined SOFA–SDC1 model improved discrimination for both AKI (AUC: 0.770) and RRT (AUC: 0.737), surpassing individual predictors. **Conclusions:** SDC1 emerged as the most reliable biomarker for assessing AKI and predicting the need for RRT, highlighting its potential role in early renal risk stratification among critically ill patients.

## 1. Introduction

Sepsis is a life-threatening syndrome resulting from a dysregulated host response to infection, frequently leading to organ dysfunction and high mortality [1]. Acute kidney injury (AKI) is a frequent complication among hospitalized patients, with an overall incidence of ~20–25% in general hospital populations and up to 47% among critically ill patients in the intensive care unit (ICU) [2]. When AKI arises in the setting of sepsis, termed sepsis-associated AKI (S-AKI), it is associated with poor clinical outcomes, including increased mortality, prolonged ICU stays, greater need for renal replacement therapy (RRT), and higher risk of chronic kidney disease [3]. Sepsis is implicated in 30–60% of all AKI cases in the ICU, and large cohort studies report S-AKI incidence rates ranging from 14% to 87% among septic patients. These findings indicate that the estimated global prevalence of S-AKI is 6–11 million cases annually, highlighting its major contribution to sepsis-related morbidity and mortality [4,5,6].

The pathophysiology of S-AKI involves multiple processes, including systemic inflammation, hemodynamic instability, microvascular dysfunction, and direct tubular injury [7]. These factors contribute to dynamic and often reversible renal impairment that may not be immediately detectable via conventional markers. Serum creatinine, the standard marker of kidney function, typically increases only after substantial renal injury and is influenced by nonrenal factors such as age, gender, and muscle mass [8]. In the context of sepsis, these limitations are further amplified, underscoring the need for earlier and more accurate biomarkers for timely diagnosis and risk stratification.

Recent studies have identified several promising biomarkers—syndecan-1 (SDC1), neutrophil gelatinase-associated lipocalin (NGAL), proenkephalin (PENK), and presepsin (PSPN)—that have shown diagnostic and prognostic potential in critical care settings, although their comparative value in S-AKI prediction and outcome assessment continues to be refined. SDC1 reflects endothelial glycocalyx degradation, which is known to occur early in sepsis and may precede functional renal impairment. NGAL has been widely studied as a tubular stress marker, and PENK and PSPN are emerging peptides involved in neuroendocrine and inflammatory modulation [9,10].

This study aimed to evaluate the predictive utility of selected biomarkers for the primary endpoints of AKI development and the need for RRT in patients with sepsis. Since biomarker sampling preceded full clinical staging of kidney injury, some patients may have already met the AKI criteria at inclusion. Nonetheless, the study focused on whether early biomarker levels could reflect current or impending renal dysfunction, support early risk stratification, and predict subsequent renal outcomes. The assessment of ICU mortality served as a secondary outcome, providing additional insight into the prognostic utility of the studied biomarkers. An improved early identification of patients at risk may facilitate timely interventions and guide individualized renal management strategies in this high-risk population.

## 2. Materials and Methods

### 2.1. Study Design and Ethical Approval

This study was designed as a prospective observational study in accordance with the Strengthening the Reporting of Observational Studies in Epidemiology (STROBE) guidelines. The study was conducted between 1 December 2021 and 30 June 2022. Ethical approval was obtained from the Clinical Research Ethics Committee of Istanbul University, Istanbul Faculty of Medicine (Approval No: 2021/1907, Approval Date: 5 November 2021). The study was registered on the national clinical trial registry (clinicaltrials.gov) with the identifier NCT05310812. All procedures involving human participants were performed in compliance with the ethical standards of the institutional and national research committees and the 1964 Declaration of Helsinki and its subsequent amendments.

### 2.2. Study Population and Grouping

Patients admitted to the ICU of Istanbul University, Istanbul Faculty of Medicine, were screened for eligibility. The study population was selected on the basis of the diagnostic criteria for sepsis as defined by the Third International Consensus Definitions for Sepsis and Septic Shock (Sepsis-3) [1]. Patients were considered to have sepsis if they met the following criteria: (i) suspected infection (defined as either the presence of a bloodstream pathogen or the initiation of antibiotic therapy) and (ii) a Sequential Organ Failure Assessment (SOFA) score ≥ 2. Patients fulfilling the inclusion and exclusion criteria were enrolled in the study. All enrolled patients received treatment in accordance with the current Surviving Sepsis Campaign guidelines [11]. Written informed consent was obtained from each patient or their legal representative, depending on the clinical status of the patient. Blood samples for both routine laboratory parameters and biomarker analysis were collected at the time of sepsis diagnosis.

#### 2.2.1. Exclusion Criteria

Patients were excluded from the study if they met any of the following criteria: (i) age under 18 years, (ii) known pre-existing chronic kidney disease and end-stage renal disease requiring dialysis, (iii) history of kidney or liver transplantation, (iv) missing demographic or laboratory data, (v) transferred from another hospital while receiving vasopressor support or mechanical ventilation (MV), (vi) brain injury and multiorgan failure following cardiopulmonary resuscitation, (vii) ICU stay less than 24 h, or (viii) terminal malignancy with no expected survival.

#### 2.2.2. Patient Grouping

Patients were categorized based on sepsis severity and clinical outcomes. Mortality status was recorded, and patients were stratified according to the Acute Physiology and Chronic Health Evaluation II (APACHE II) score (<20 vs. ≥20) and SOFA score (<9 vs. ≥9) [12]. AKI was assessed by the Kidney Disease: Improving Global Outcomes (KDIGO) criteria during the first 7 days of the ICU stay [13,14]. AKI was defined as the presence of at least one of the following: (i) a rise in serum creatinine to 1.5 times the baseline level upon ICU admission, (ii) an increase in serum creatinine by >0.3 mg/dL within 48 h, (iii) a measured serum creatinine >1.5 mg/dL in patients with unknown baseline values, or (iv) a urine output <0.5 mL/kg/h for 6–12 h, as specified by the KDIGO definition for stage 1 AKI. At the time of inclusion, some patients already fulfilled the AKI criteria; however, this was not considered exclusionary. The primary objective was to determine whether early biomarker levels—measured at the time of sepsis diagnosis—could predict the presence, severity, or progression of AKI, as well as the subsequent need for RRT and likelihood of renal recovery. Decisions regarding fluid management and the need for RRT (Fresenius Medical Care—multiFiltratePRO) were made by attending intensivists according to standard ICU protocols and who were blinded to the study, with all RRT initiations recorded prospectively and occurring after biomarker sampling. Renal nonrecovery was defined as the persistence of any stage of AKI within 7 days, ongoing RRT on day 7, or death occurring within 7 days after AKI diagnosis. On the basis of these assessments, patients were further classified as AKI vs. non-AKI, RRT-positive vs. RRT-negative, and renal recovery-positive vs. renal recovery-negative.

### 2.3. Data Collection

For all patients included in the study, demographic data (age, gender, and body mass index (BMI)) and clinical characteristics (reasons for admission, patient category [medical: non-operative conditions such as pneumonia, urinary tract infection or heart failure; surgical: postoperative patients admitted following elective or emergency operations], comorbidities, Charlson Comorbidity Index (CCI), Glasgow Coma Scale (GCS), and laboratory parameters (white blood cell count (WBC, 10^3^/µL), neutrophil count (10^3^/µL), lymphocyte count (10^3^/µL), serum C-reactive protein level (CRP, mg/L), procalcitonin concentration (PCT, ng/mL), serum lactate level (mmol/L), serum creatinine level (mg/dL), serum albumin level (g/dL), N-terminal pro-B-type natriuretic peptide (NT-proBNP, µg/L), and D-dimer level (µg/L)) were recorded. During ICU follow-up, the need for MV and vasopressor support was assessed and documented on a daily basis.

### 2.4. Biomarker Sampling and Analysis

**Figure 1:**
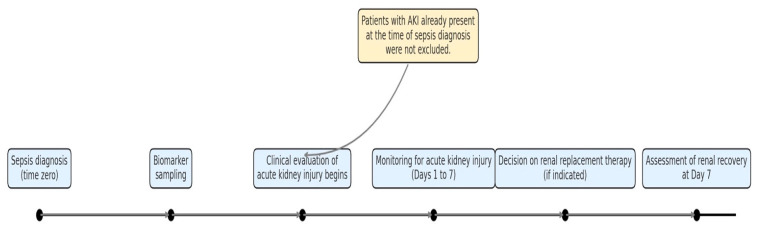
Timeline of clinical and biomarker-based assessments in septic ICU patients. Blood sampling for biomarker analysis was performed immediately at the time of sepsis diagnosis (time zero). Clinical evaluation of AKI followed, with monitoring conducted over the first 7 days of ICU admission. RRT decisions were made based on standard clinical criteria, and renal recovery was assessed on day 7. The timeline illustrates the temporal relationship between biomarker collection and clinical outcomes. Note: Patients who already fulfilled the AKI criteria at the time of sepsis diagnosis were not excluded from the study, as the analysis aimed to assess the predictive and stratification value of early biomarker levels regardless of initial renal function.

Upon enrollment, two plain blood tubes (5 mL each) were collected from each patient for biomarker analysis (Figure 1). The samples were centrifuged at 3500 RPM for 10 min to separate the serum, which was then aliquoted into 2 mL Eppendorf tubes and stored at –80 °C under appropriate conditions until analysis. At the end of the sample collection period, the following biomarkers were biochemically analyzed via an enzyme-linked immunosorbent assay (ELISA), following the manufacturers’ protocols: (i) PSPN (BTLAB—E3745Hu—Human Presepsin ELISA Kit), (ii) NGAL (ELAB—E-EL-H0096—Human NGAL ELISA Kit), (iii) SDC1 (ELAB—E-EL-H1298—Human Syndecan-1 ELISA Kit), and (iv) PENK (BTLAB—E2341Hu—Human Proenkephalin ELISA Kit).

### 2.5. Sample Size Calculation

The sample size was calculated to evaluate the predictive performance of a novel biomarker for RRT requirements in patients with sepsis. On the basis of previous studies, the incidence of RRT among septic patients was estimated to be approximately 20%. Assuming an area under the receiver operating characteristic curve (AUC) of 0.70 for the biomarker, with a significance level (α) of 0.05 and a statistical power (1−β) of 0.80, a total sample size of 89 patients would be sufficient to detect meaningful discrimination. To account for potential missing data and ensure adequate power, a total of 140 patients were planned to be enrolled.

### 2.6. Statistical Analysis

All the statistical analyses were performed via IBM SPSS Statistics software (version 25; Chicago, IL, USA). The distribution of continuous variables was assessed via the Kolmogorov–Smirnov test and graphical methods. Continuous variables with a normal distribution are expressed as the mean ± standard deviation, whereas non-normally distributed variables are presented as the median and interquartile range (IQR) (median (Q1–Q3)). Categorical variables are summarized as counts and percentages (n, %). For comparisons between two groups, Student’s t test was used for normally distributed continuous variables, and the Mann–Whitney U test was used for non-normally distributed variables. Categorical variables were compared via the chi-square test or Fisher’s exact test, as appropriate. Variables that showed statistical significance in the univariate analysis were included in a multivariate logistic regression model in which the enter method was used to identify independent predictors and their odds ratios (ORs). Receiver operating characteristic (ROC) curve analysis was conducted to determine the predictive performance of the scoring systems, and the AUC, sensitivity, and specificity values were calculated. A *p* value < 0.05 was considered statistically significant.

## 3. Results

### 3.1. Study Population and Flow Diagram

**Figure 2:**
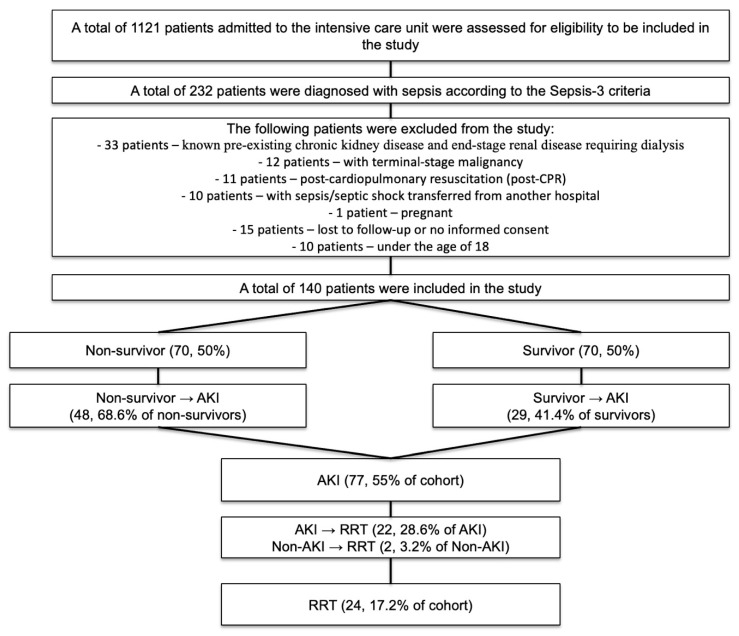
Flow diagram of study patients.

A total of 1121 patients were screened for eligibility, 140 of whom met the inclusion criteria and were enrolled in the final cohort. Among these, 70 patients (50%) survived, and 70 (50%) died during their hospital stay. AKI occurred in 77 patients (55% of the cohort), and RRT was administered to 24 patients (17.2%). (Figure 2)

### 3.2. Comparison of Clinical and Laboratory Characteristics According to AKI and RRT Status

Patients who developed AKI were significantly older than the non-AKI group (67.8 ± 13.4 vs. 62.0 ± 16.0 years, *p* = 0.036). Respiratory comorbidities (44.2% vs. 25.4%, *p* = 0.021) and gastrointestinal comorbidities (21.4% vs. 7.9%, *p* = 0.015) were also more prevalent in the AKI group. Disease severity scores were significantly greater in the AKI group than in the non-AKI group, as reflected by the APACHE II score [18.0 (11.5–26.0) vs. 13.0 (9.0–18.0), *p* < 0.001] and SOFA score [8.0 (6.0–10.0) vs. 5.0 (4.0–7.0), *p* < 0.001]. In terms of laboratory parameters, patients with AKI had significantly higher CRP [189.1 (115.2–294.6) vs. 155.0 (49.0–257.0) mg/L, *p* = 0.043], PCT [4.1 (0.87–36.8) vs. 1.38 (0.98–7.7) ng/mL, *p* = 0.007], and NT-proBNP [6252 (2032–15,701) vs. 2242 (542–8416) µg/L, *p* = 0.004] levels.

In the comparison between RRT (+) and RRT (–) patients, no statistically significant differences were found in age, comorbidities, or most laboratory parameters. A borderline significant difference was observed in initial SOFA scores [8.0 (5.0–10.0) vs. 7.0 (5.0–9.0), *p* = 0.050], with higher scores among patients who required RRT. Other variables—including gender, BMI, admission category, MV, and several inflammatory markers—did not differ significantly between the groups (Table 1).

### 3.3. Serum Biomarker Levels Stratified by Clinical Outcomes and Disease Severity

Among patients stratified by SOFA score, those with a SOFA score ≥ 9 had significantly higher SDC1 levels [4.65 (2.96–7.92) vs. 3.50 (2.36–6.05) ng/mL, *p* = 0.032] and markedly elevated NGAL levels [7.48 (4.19–10.5) vs. 4.57 (2.19–7.34) ng/mL, *p* < 0.001] than those with a SOFA score ≤ 9. Similarly, both SDC1 and NGAL levels were significantly greater in patients who developed AKI [*p* < 0.001 for both] and in those who required RRT [SDC1: 6.89 (3.98–8.28) vs. 3.50 (2.32–6.13), *p* < 0.001]. No statistically significant differences were observed in biomarker levels when patients were stratified by in-hospital mortality rates or APACHE II scores. PENK and PSPN levels also did not significantly differ across any of the subgroups analyzed. In patients diagnosed with AKI, no statistically significant differences were observed in the serum levels of SDC1, NGAL, PENK, or PSPN between those who achieved renal recovery within 7 days and those who did not (*p* > 0.05 for all comparisons). Although the median values were slightly greater in the nonrecovery group, these differences did not reach statistical significance (Table 2).

### 3.4. Multivariate Logistic Regression Analysis of AKI and RRT Initiation Risk Factors in Sepsis Patients

For AKI evaluation, two variables emerged as statistically significant predictors. Higher initial SOFA scores were independently associated with an increased risk of AKI (OR: 1.258; 95% CI: 1.047–1.511; *p* = 0.014). Additionally, elevated levels of SDC1 were significantly associated with AKI occurrence (OR: 1.201; 95% CI: 1.025–1.406; *p* = 0.024). With respect to RRT initiation, SDC1 was the only independent predictor (OR: 1.260; 95% CI: 1.078–1.472; *p* = 0.004), whereas the initial SOFA score did not reach statistical significance (*p* = 0.122). None of the other clinical or biochemical variables were found to be statistically significant predictors in the multivariable models (Table 3).

### 3.5. Receiver Operating Curve Analysis of Biomarkers for AKI Evaluation and RRT Initiation

**Figure 3:**
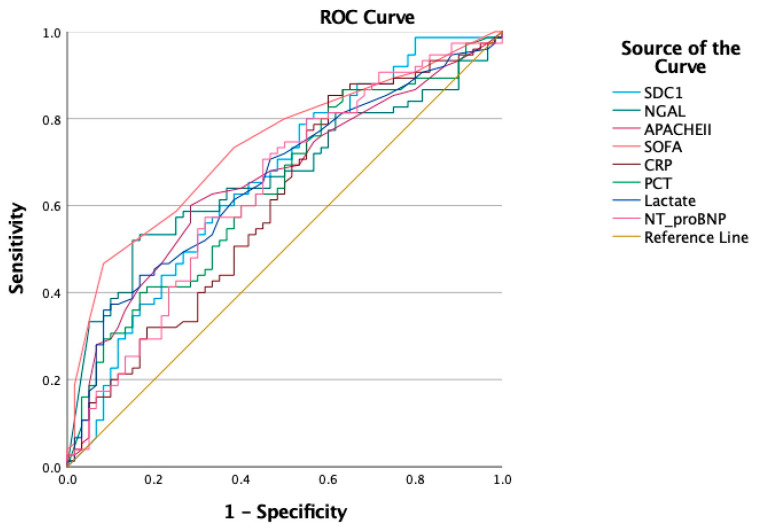
Receiver operating characteristic curve analysis for AKI.

**Figure 4:**
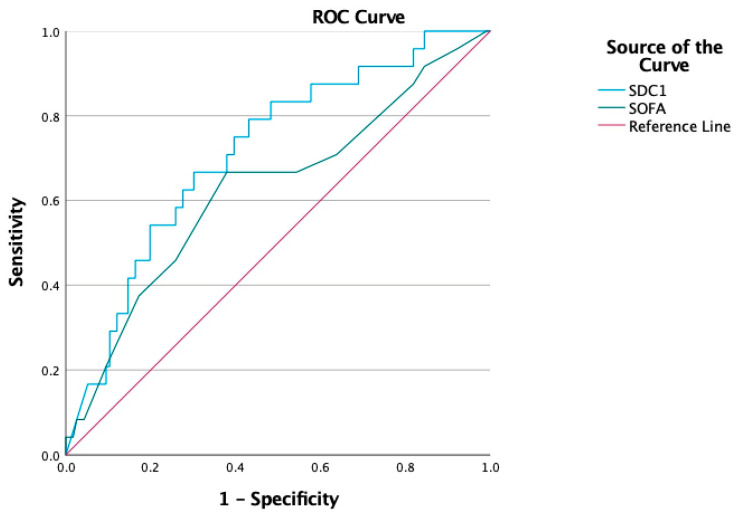
Receiver operating characteristic curve analysis for RRT.

**Figure 5:**
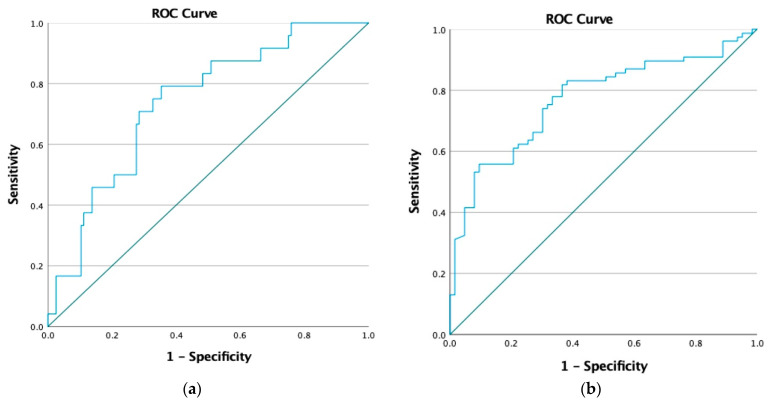
Receiver operating characteristic curve analysis of the SDC1-SOFA model for AKI evaluation and RRT prediction. (**a**) SDC1-SOFA clinical model for AKI evaluation, AUC: 0.770 (0.692–0.849), *p* < 0.001; (**b**) SDC1-SOFA clinical model for RRT initiation, AUC: 0.737 (0.635–0.838), *p* < 0.001.

For AKI evaluation (Figure 3), the highest AUC was observed for the initial SOFA score (AUC: 0.736; 95% CI: 0.653–0.820; *p* < 0.001), with a cutoff of 8.5, sensitivity of 0.467, and specificity of 0.920. Statistically significant AUC values were also observed for SDC1 (AUC: 0.659, *p* = 0.002), NGAL (AUC: 0.666, *p* = 0.001), the APACHE II score (AUC: 0.659, *p* = 0.002), lactate (AUC: 0.660, *p* = 0.001), CRP (AUC: 0.606, *p* = 0.035), PCT (AUC: 0.639, *p* = 0.006), and NT-proBNP (AUC: 0.640, *p* = 0.005). For RRT initiation (Figure 4), SDC1 demonstrated a statistically significant AUC of 0.715 (95% CI: 0.606–0.824; *p* = 0.001), with a cutoff of 5.32. The AUC for the initial SOFA score did not reach statistical significance (AUC: 0.626; *p* = 0.052) (Table 4). For AKI evaluation (Figure 5a), the combined SDC1-SOFA model yielded an AUC of 0.770 (95% CI: 0.692–0.849; *p* < 0.001). For RRT initiation (Figure 5b), the same model had an AUC of 0.737 (95% CI: 0.635–0.838; *p* < 0.001).

### 3.6. Univariable and Multivariable Analysis of Mortality-Associated Clinical and Laboratory Variables in Sepsis

There were no significant differences in age, gender, or BMI between the two groups. The reason for ICU admission significantly differed between the groups (*p* = 0.023). A significantly greater proportion of nonsurvivors than survivors were categorized as medical patients (61.4% vs. 35.7%, *p* = 0.002). Respiratory comorbidities were significantly more common in nonsurvivors (47.1% vs. 24.3%, *p* = 0.005). The other comorbidities were not significantly different. Disease severity was markedly greater among nonsurvivors, as evidenced by higher APACHE II scores [18.0 (12.8–23.3) vs. 12.0 (10.0–18.3), *p* = 0.007], higher SOFA scores [8.0 (5.0–10.0) vs. 7.0 (5.0–8.0), *p* = 0.012], and lower GCS scores at admission [10 (8–13) vs. 13 (10–13), *p* < 0.001]. Nonsurvivors had longer ICU stays (median 9 vs. 7 days, *p* = 0.036), were more likely to require MV (98.6% vs. 74.3%, *p* < 0.001), and had longer MV durations (*p* < 0.001). The need for vasopressor support and duration (*p* = 0.005, *p* < 0.001, respectively), AKI incidence (68.6% vs. 41.4%, *p* = 0.001), and RRT requirement (27.1% vs. 7.1%, *p* = 0.002) were significantly greater in the nonsurvivors. NT-proBNP levels were significantly elevated in nonsurvivors [7566 µg/L (1885–18,027)] compared to survivors [2617 µg/L (651–8204); *p* = 0.004]. Other laboratory parameters, including WBC count, serum CRP level, procalcitonin concentration, serum creatinine level, serum lactate level, NLR, PLR, serum albumin level, and D-dimer level, did not differ significantly between the groups. Independent predictors of ICU mortality included medical patient categorization (OR: 3.014; 95% CI: 1.217–7.466; *p* = 0.017), the presence of respiratory comorbidities (OR: 3.001; 95% CI: 1.139–7.907; *p* = 0.026), and lower GCS scores at admission (OR: 0.737; 95% CI: 0.595–0.914; *p* = 0.005). Other variables, including APACHE II and SOFA scores, MV, vasopressor support, AKI, RRT, and NT-proBNP levels, did not retain statistical significance in the multivariable model (Table 5).

## 4. Discussion

This prospective observational study evaluated the prognostic and diagnostic utility of novel biomarkers focusing on their associations with disease severity, the development of S-AKI, the need for RRT, and ICU mortality in critically ill septic patients. Among these, SDC1 was the only independent predictor of both AKI onset (OR: 1.201; 95% CI: 1.025–1.406; *p* = 0.024) and RRT initiation (OR: 1.260; 95% CI: 1.078–1.472; *p* = 0.004), whereas NGAL retained significance only in univariable analyses. PENK and PSPN did not discriminate any prespecified outcomes. In the ROC analysis, the combined SDC1-SOFA model achieved the highest AUC values for both AKI evaluation (AUC: 0.770; 95% CI: 0.692–0.849) and RRT initiation (AUC: 0.737; 95% CI: 0.635–0.838), outperforming individual biomarkers, including SDC1 alone (AUC: 0.659 for AKI, 0.715 for RRT), NGAL (AUC: 0.666 for AKI), PENK (AUC: 0.600 for AKI), and PSPN (AUC: 0.588 for AKI), across all evaluated endpoints.

Among the biomarkers analyzed, SDC1 demonstrated the strongest and most consistent predictive capacity. As a component of the endothelial glycocalyx, SDC1 is shed during systemic inflammation and vascular stress, contributing to organ dysfunction [15,16]. Elevated SDC1 levels were significantly associated with AKI, RRT need, and higher SOFA scores (>9), indicating its correlation with both renal and overall organ dysfunction, and our study identified SDC1 as the strongest predictor of RRT (AUC: 0.715) and a significant marker for AKI (AUC: 0.659). The superior performance of the SDC1-SOFA composite model in ROC analysis further reinforces its prognostic relevance. These findings are consistent with previous ICU studies, including those by Huang et al., which linked SDC1 with APACHE II and SOFA scores and showed AUCs of up to 0.885 for the prediction of septic shock [16]. Prior studies have linked high SDC1 levels to renal dysfunction in sepsis: in a meta-analysis of 11 studies, patients with AKI had significantly higher SDC1 levels compared to those without [17]. SDC1 has also shown predictive validity in nonseptic cohorts, such as those with postoperative AKI following cardiac surgery, with reported AUCs between 0.77 and 0.84 [18,19]. Our results, therefore, support the emerging view that SDC1 is a robust marker of endothelial injury with broad applicability across critical care contexts.

NGAL, a marker of tubular stress and injury, was significantly elevated in septic patients who developed AKI. Prior research has shown that NGAL levels increase before the serum creatinine level and predict AKI with high diagnostic accuracy, with AUC values ranging from 0.78 to 0.92 depending on the sample type (plasma, serum, or urine) and timing [20,21,22,23]. However, while NGAL showed statistical significance in univariable analysis, it failed to demonstrate independent predictive power in adjusted models, possibly reflecting both its susceptibility to systemic inflammatory responses and the inclusion of patients with established AKI at the time of sepsis recognition, which may have confounded its prognostic discrimination [24]. NGAL has also been linked to sepsis severity and mortality in prior studies, suggesting that it may serve best as a complementary biomarker within a broader panel rather than as a standalone predictor, particularly when it is interpreted alongside organ dysfunction scores and endothelial injury markers such as SDC1 [23,24].

Although PSPN has been proposed as a promising biomarker for early AKI detection and mortality risk stratification in sepsis patients, our study revealed no significant associations between PSPN levels and AKI development, RRT requirements, or mortality. This finding contrasts with prior findings by Kim et al., who reported a progressive increase in PSPN levels across AKI stages and demonstrated an AUC of 0.793 (sensitivity: 77.0%, specificity: 81.7%) for AKI prediction in septic patients [25]. With respect to mortality, the prognostic value of PSPN remains controversial. Some studies reported significantly elevated PSPN concentrations among nonsurvivors, with AUCs ranging from 0.636 to 0.799 for mortality prediction [26,27,28]. Conversely, other cohorts—including our own—did not observe a significant difference in PSPN levels between survivors and nonsurvivors [29,30]. These discrepancies may be attributed to variations in disease severity, the timing of biomarker measurement, and the influence of renal clearance, since PSPN is excreted via the kidneys and may accumulate independently of ongoing injury [25,30].

PENK, which represents both glomerular filtration and tubular injury, similarly failed to differentiate patients by AKI status, RRT requirement, or mortality in our analysis. These findings are in line with those of Verras et al., who also reported no association between PENK and AKI in septic shock patients [31]. A meta-analysis by Lin et al. reported moderate diagnostic utility of PENK (AUC ~0.77), but its specificity was reduced in patients with cardiovascular disease and diabetes—both common comorbidities in our cohort [32,33]. While some studies have reported that PENK is predictive of mortality, with AUCs greater than 0.72 and high sensitivity, our data did not replicate this prognostic value [31]. These discrepancies highlight the heterogeneity of the clinical performance of PENK and the need for further external validation in diverse ICU populations.

In this analysis of septic ICU patients, none of the evaluated biomarkers demonstrated a significant association with renal recovery within 7 days among patients who developed AKI. These findings align with recent findings indicating that commonly studied biomarkers such as NGAL have a limited ability to distinguish transient AKI from persistent AKI. In a recent meta-analysis, NGAL showed modest diagnostic accuracy for predicting persistent AKI, with a diagnostic odds ratio of 4 and specificity below 0.70. While biomarkers such as PENK have shown promise in selected populations, their role in predicting short-term renal reversibility remains uncertain in the context of heterogeneous septic ICU patients [34].

In our cohort, 20.7% of ICU admissions fulfilled the Sepsis-3 criteria, aligning with prior reports from high-income countries where sepsis accounted for 20–30% of ICU admissions [5,35]. This finding underscores the ongoing burden of sepsis in tertiary critical care settings. The ICU mortality rate among septic patients in our study was 50%, exceeding the 25–45% range reported in previous cohorts and likely reflecting greater illness severity [36,37]. Consistent with the established literature, nonsurvivors had significantly higher APACHE II and SOFA scores and lower GCS scores at admission [38,39]. Mortality was also more common among patients with pulmonary or gastrointestinal comorbidities and those admitted from medical wards, as previously reported [40,41]. In contrast, age, sex, and baseline inflammatory markers (CRP and PCT), which are commonly reported predictors of sepsis mortality, were not significantly associated with outcomes in our cohort [42,43]. These findings are in line with recent evidence suggesting the limited prognostic utility of CRP and PCT in isolation and may reflect cohort-specific characteristics, the timing of biomarker measurement, and sepsis heterogeneity [44].

A substantial proportion of patients (5′%) developed S-AKI, with 17.2% requiring RRT—which is consistent with previously reported incidence ranges of 14–87% for S-AKI and 3.6–40% for RRT utilization in critically ill septic populations [6,45,46,47]. Mortality among S-AKI patients reached 68.6% and rose to 79.2% in those receiving RRT, whereas it reached 44.0% in those not requiring RRT (*p* = 0.003), which aligns with literature estimates ranging from 11% to 77% [6,48,49]. These broad variations reflect heterogeneity in diagnostic and management strategies across ICUs. Notably, the SOFA score independently predicts S-AKI development, in line with previous data highlighting the prognostic relevance of nonrenal SOFA components [3]. While traditional risk factors such as age, diabetes, and vasopressor use were not statistically significant in our model, this may reflect differences in timing, sample size, or population characteristics [48,50]. Despite representing a high-risk group, our cohort remains broadly comparable to international ICU populations in terms of AKI and RRT burden.

This study has several notable strengths. Its prospective design, the application of standardized and validated definitions (Sepsis-3 for sepsis and KDIGO for AKI), and the use of uniform, time-specific biomarker sampling enhance methodological rigor. Furthermore, the simultaneous evaluation of four biomarkers representing distinct renal pathophysiological mechanisms offers potential translational insight into early kidney injury in sepsis. However, certain limitations should be acknowledged. The single-center design may limit generalizability, and the modest sample size—especially for RRT analysis—reduces statistical power. Single-time-point sampling precludes dynamic assessment. Additionally, as patients were enrolled at the time of sepsis diagnosis without excluding those with pre-existing AKI, biomarker levels may reflect established injury rather than predict future AKI. While this reflects real-world clinical practice, it limits strict interpretation of predictive performance. Fibrinogen levels were not routinely available and could not be included in the inflammatory marker analysis. Consequently, albumin/fibrinogen and CRP/fibrinogen ratios were not calculated, which may represent a limitation in assessing the full inflammatory burden. While no single biomarker provides comprehensive prognostic utility, endothelial injury markers such as SDC1 may offer added value in guiding renal monitoring, triaging high-risk patients, and informing RRT timing decisions. Future studies should prioritize the development of multimarker panels and dynamic assessment protocols that reflect the complex and evolving nature of S-AKI. Additionally, biomarker-guided management strategies and interventional trials are needed to determine whether early identification of renal risk can translate into improved clinical outcomes.

## 5. Conclusions

Consequently, in this prospective observational study of critically ill septic patients, SDC1 demonstrated the strongest and most consistent predictive value and was independently associated with both S-AKI and the need for RRT. While NGAL showed univariable associations with AKI, it did not retain significance in the multivariable analysis. Neither PENK nor PSPN provided added prognostic utility. Additionally, higher SOFA scores were independently linked to AKI, and the combined SOFA-SDC1 model yielded the highest predictive performance for both AKI evaluation and RRT initiation, outperforming individual biomarkers. These findings underscore the clinical potential of integrating endothelial biomarkers with established clinical scores to enhance early renal risk stratification in septic ICU patients. Future multicenter studies with larger sample sizes and longitudinal biomarker profiling are warranted to confirm these results and explore their applicability in clinical decision-making.

## Figures and Tables

**Table 1:** Clinical variables and laboratory data of the cohort comparing AKI vs. non-AKI patients and RRT-positive vs. RRT-negative patients.

Parameter	AKI (77, 55%)	Non-AKI (63, 45%)	*p*	RRT-Positive (24, 17.2%)	RRT-Negative (116, 82.8%)	*p*
**Clinical variables**						
**Age (years)**	67.8 ± 13.4	62 ± 1.6	**0.036**	65.3 ± 12.8	65.2 ± 16.8	0.961
**Gender (Male)**	52, 67.5%	36, 57.1%	0.206	15, 62.5%	73, 62.9%	0.968
**BMI (kg/m^2^)**	30.7 (26.2–33.2)	29.7 (24.8–32.3)	0.186	26.6 (23.6–32.3)	30.3 (26.2–33.1)	0.137
**Reason for admission** ***Abdominal*** ***Respiratory*** ***Soft tissue*** ***Urinary*** ***Endocarditis*** ***Neurological***	37, 48.1% 33, 42.9% 4, 5.2% 1, 1.3% 2, 2.6% 0, 0%	30, 47.6% 28, 44.4% 2, 3.2% 1, 1.6% 0, 0% 2, 3.2%	0.486	11, 45.8% 12, 50.0% 1, 4.2% 0, 0% 0, 0% 0, 0%	56, 48.2% 49, 42.2% 5, 4.3% 2, 1.7% 2, 1.7% 2, 1.7%	0.908
**Patient category** ***Surgical*** ***Medical***	41, 53.2% 36, 46.8%	31, 49.2% 32, 50.8%	0.634	12, 50% 12, 50%	60, 51.7% 56, 48.2%	0.878
**Comorbidities** ***Cardiac*** ***Respiratory*** ***Neurologic*** ***Endocrinology*** ***Gastrointestinal*** ***Malignancy***	52, 67.5% 34, 44.2% 20, 26% 34, 44.2% 15, 19.5% 29, 37.3%	36, 57.1% 16, 25.4% 17, 27% 21, 33.3% 24, 38.1% 30, 47.6%	0.206 **0.021** 0.893 0.192 **0.015** 0.235	16, 66.7% 11, 45.8% 5, 20.8% 11, 45.8% 4, 16.7% 8, 33.3%	72, 37.9% 39, 33.6% 32, 27.6% 44, 37.9% 35, 30.2% 51, 44%	0.671 0.349 0.495 0.471 0.179 0.373
**CCI**	6.0 (4.0–8.5)	5.0 (3.0–7.0)	0.066	5.0 (3.3–8.8)	6.0 (3.0–8.0)	0.870
**APACHE II score (on the first day of admission)**	18.0 (11.5–26.0)	13.0 (9.0–18.0)	**<0.001**	14.0 (10.5–21.3)	15.5 (10.0–22.0)	0.750
**GCS**	10.0 (8.0–13.0)	11.5 (10.0–13.0)	0.054	10.0 (8.3–11.8)	11.0 (9.0–13.0)	0.084
**MV**	69, 89.6%	52, 82.5%	0.224	23, 95.8%	98, 84.5%	0.197
**Vasopressor support**	70, 90.9%	59, 93.7%	0.754	22, 91.7%	107, 92.2%	1000
**The initial SOFA score**	8.0 (6.0–10.0)	5.0 (4.0–7.0)	**<0.001**	8.0 (5.0–10.0)	7.0 (5.0–9.0)	**0.05**
**Mortality**	48, 62.3%	22, 34.9%	**0.001**	19, 79.2%	51, 44.0%	**0.003**
**Laboratory data**						
**WBC count (10^3^/µL)**	12.1 (7.8–16.6)	10.3 (7.1–16.5)	0.432	11.5 (8.1–17.5)	11.7 (7.4–16.1)	0.849
**Neutrophil count (10^3^/µL)**	10.6 (6.3–15.3)	9.2 (5.4–14.5)	0.312	10.3 (5.9–15.8)	9.8 (6.0–14.7)	0.774
**Lymphocyte count (10^3^/µL)**	0.7 (0.4–1.3)	0.8 (0.5–1.1)	0.534	0.6 (0.3–1.2)	0.8 (0.5–1.1)	0.327
**NLR**	12.8 (6.7–23.5)	12.2 (6.1– 21.0)	0.419	17.8 (6.3–37.5)	12.4 (6.3–21.2)	0.249
**TLR**	251.6 (114.9–563.1)	376.7 (202.5–538.0)	0.125	245.0 (87.8–703.3)	332.5 (157.2–516.7)	0.854
**CRP (mg/L)**	198.1 (115.2–294.6)	155.0 (49.0–257.0)	**0.043**	226.5 (114.8–342.5)	173.5 (65.5–259.3)	0.077
**PCT (ng/mL)**	4.1 (0.87–36.8)	1.4 (0.38–7.7)	**0.007**	2.48 (0.6–50.9)	3.3 (0.6, 14.9)	0.707
**Albumin (g/dL)**	2.74 (2.48–3.1)	2.71 (2.5–3.3)	0.755	2.89 (2.53–3.43)	2.71 (2.47–3.12)	0.263
**Lactate (mmol/L)**	2.2 (1.5–4.8)	1.6 (1.1–2.4)	**0.003**	2.0 (1.5–4.5)	1.9 (1.2–3.4)	0.306
**NT-proBNP (µg/L)**	6252 (2032–15,701)	2242 (542–8416)	**0.004**	7545 (1203–13,907)	4408 (1186–10,704)	0.465
**D-dimer (µg/L)**	2385 (1038–5829)	1482 (884–4144)	0.182	2340 (1038–6046)	1640 (950–4649)	0.545

AKI, acute kidney injury; RRT, renal replacement therapy; BMI, body mass index; CCI, Charlson Comorbidity Index; APACHE, Acute Physiologic and Chronic Health Evaluation; GCS, Glasgow Coma Scale; SOFA, Sequential Organ Failure Assessment; ICU, intensive care unit; MV, mechanical ventilation; WBC, white blood cell count; NLR, neutrophil–lymphocyte ratio; PLR, platelet–lymphocyte ratio; CRP, C-reactive protein; PCT, procalcitonin; NT-proBNP, N-terminal pro-B-type natriuretic peptide.

**Table 2:** Comparison of serum biomarker levels.

Mortality			
	Nonsurvivor	Survivor	*p*
**SDC1** **(ng/mL)**	3.85 (2.37–7.09)	3.83 (2.63–6.76)	0.891
**NGAL** **(ng/mL)**	5.38 (2.88–8.44)	5.33 (2.37–8.22)	0.678
**PENK** **(ng/L)**	361.9 (321.1–470.2)	357.4 (306.9–440.6)	0.671
**PSPN** **(ng/L)**	131.9 (107.1–154.0)	126.3 (97.8–159.5)	0.780
**Severity/1**			
	**APACHE < 20 (101, 72.1%)**	**APACHE > 20 (39, 27.9%)**	***p***
**SDC1** **(ng/mL)**	3.83 (2.38–6.54)	3.92 (2.74–7.47)	0.492
**NGAL** **(ng/mL)**	5.02 (2.32–7.66)	6.22 (3.38–10.5)	0.075
**PENK** **(ng/L)**	353.8 (310.2–441.3)	363.9 (322.9–471.5)	0.468
**PSPN** **(ng/L)**	125.6 (98.2–152.4)	136.7 (111.4–162.1)	0.109
**Severity/2**			
	**SOFA < 9 (99, 70.7%)**	**SOFA > 9 (41, 29.3%)**	***p***
**SDC1** **(ng/mL)**	3.50 (2.36–6.05)	4.65 (2.96–7.92)	**0.032**
**NGAL** **(ng/mL)**	4.57 (2.19–7.34)	7.48 (4.19–10.5)	**<0.001**
**PENK** **(ng/L)**	358.9 (306–424.7)	359.4 (325.6–493.3)	0.266
**PSPN** **(ng/L)**	128.5 (99.5–152.8)	131.2 (103.8–167.0)	0.438
**AKI evaluation**			
	**AKI**	**Non-AKI**	***p***
**SDC1** **(ng/mL)**	5.01 (2.94–7.87)	3.13 (1.83–5.17)	**<0.001**
**NGAL** **(ng/mL)**	7.22 (3.12–10.5)	4.15 (2.14–6.5)	**<0.001**
**PENK** **(ng/L)**	350.6 (313.9–462.1)	368.8 (311.5–440.7)	0.859
**PSPN** **(ng/L)**	131.2 (104.8–159.5)	125.7 (98.2–147.5)	0.425
**RRT initiation**			
	**RRT-positive**	**RRT-negative**	***p***
**SDC1** **(ng/mL)**	6.89 (3.98–8.28)	3.50 (2.32–613)	**<0.001**
**NGAL** **(ng/mL)**	6.22 (1.21–10.5)	5.38 (2.56–8.14)	0.957
**PENK** **(ng/L)**	344.7 (293.2–493.3)	362.2 (315.2–440.7)	0.580
**PSPN** **(ng/L)**	122.4 (103.7–152.5)	130.3 (102.1–158.0)	0.752
**Renal recovery (for AKI patients)**			
	**Renal recovery-positive (30, 39.0%)**	**Renal recovery-negative (47, 61.0%)**	***p***
**SDC1 (ng/mL)**	4.77 (3.27–7.89)	5.16 (2.87–7.89)	0.766
**NGAL (ng/mL)**	7.29 (3.66–10.5)	6.28 (2.93–10.5)	0.361
**PENK (ng/L)**	340.3 (304.0–425.2)	361.2 (327.5–486.5)	0.185
**PSPN (ng/L)**	127.4 (97.1–164.1)	132.7 (108.3–157.3)	0.884

SDC1, syndecan-1; NGAL, neutrophil gelatinase-associated lipocalin; PENK, proenkephalin; PSPN, presepsin; APACHE, Acute Physiologic and Chronic Health Evaluation; SOFA, Sequential Organ Failure Assessment; AKI, acute kidney injury; RRT, renal replacement therapy.

**Table 3:** Multivariate logistic regression analysis of AKI and RRT initiation risk factors in sepsis patients.

AKI Evaluation		
Parameter	OR (%95, CI)	*p*
**Comorbidities, *Respiratory***	0.482 (0.184–1.262)	0.137
**Comorbidities, *Gastrointestinal***	1.979 (0.726–5.392)	0.182
**APACHE II score (on the first day of admission)**	1.047 (0.985–1.113)	0.142
**The initial SOFA score**	1.258 (1.047–1.511)	**0.014**
**Age (years)**	1.022 (0.994–1.051)	0.118
**CRP (mg/L)**	1.003 (1.000–1.007)	0.079
**PCT (ng/mL)**	1.010 (0.992–1.029)	0.270
**Lactate (mmol/L)**	1.158 (0.939–1.427)	0.171
**NT-proBNP (µg/L)**	1 (1–1)	0.389
**SDC1 (ng/mL)**	1.201 (1.025–1.406)	**0.024**
**NGAL (ng/mL)**	1.120 (0.967–1.299)	0.131
*p* < 0.001, Nagelkerke R Square: 0.421		
**RRT initiation**		
**The initial SOFA score**	1.133 (0.967–1.328)	0.122
**SDC1 (ng/mL)**	1.260 (1.078–1.472)	**0.004**
*p*:0.002, Nagelkerke R Square: 0.147		

AKI, acute kidney injury; APACHE, Acute Physiologic and Chronic Health Evaluation; SOFA, Sequential Organ Failure Assessment; PCT, procalcitonin; SDC1, syndecan-1; NGAL, neutrophil gelatinase-associated lipocalin; NT-proBNP, N-terminal pro-B-type natriuretic peptide; RRT, renal replacement therapy.

**Table 4:** Receiver operating characteristic curve analysis.

Parameter	Cutoff	AUC (95% CI)	Sensitivity	Specificity	*p*	Youden Index
**AKI evaluation**						
**SDC1 (ng/mL)**	2.73	0.659 (0.566–0.752)	0.800	0.450	**0.002**	0.250
**NGAL (ng/mL)**	7.08	0.666 (0.575– 0.758)	0.520	0.850	**0.001**	0.370
**APACHE II score (on the first day of admission)**	16.5	0.659 (0.567–0.751)	0.600	0.720	**0.002**	0.320
**The initial SOFA score**	8.5	0.736 (0.653–0.820)	0.467	0.920	**<0.001**	0.387
**CRP (mg/L)**	72.9	0.606 (0.509–0.702)	0.853	0.400	**0.035**	0.253
**PCT (ng/mL)**	10.8	0.639 (0.546–0.732)	0.400	0.833	**0.006**	0.233
**Lactate (mmol/L)**	3.7	0.660 (0.568–0.752)	0.360	0.917	**0.001**	1.277
**NT-proBNP (µg/L)**	5737	0.640 (0.545–0.735)	0.547	0.700	**0.005**	0.247
**RRT initiation**						
**SDC1 (ng/mL)**	5.32	0.715 (0.606–0.824)	0.667	0.698	**0.001**	0.365
**The initial SOFA score**	7.5	0.626 (0.496–0.757)	0.667	0.621	0.052	0.288

AUC, area under the curve: AKI, acute kidney injury; RRT, renal replacement therapy; SDC1, syndecan-1; NGAL, neutrophil gelatinase-associated lipocalin; APACHE, Acute Physiologic and Chronic Health Evaluation; SOFA, Sequential Organ Failure Assessment; CRP, C-reactive protein; PCT, procalcitonin; NT-proBNP, N-terminal pro-B-type natriuretic peptide.

**Table 5:** Comparison of clinical and laboratory characteristics between survivors and nonsurvivors with multivariate analysis of mortality predictors.

Parameter	Nonsurvivor (70, 50%)	Survivor (70, 50%)	*p*	OR (%95 CI)	*p*
**Clinical variables**					
**Age (years)**	65.8 ± 15.6	64.6 ± 16.8	0.654		
**Gender (Male)**	42, 60%	46, 65.7%	0.484		
**BMI (kg/m^2^)**	29.7 (24.7–33.2)	30.1 (26.5–32.5)	0.882		
**Reason for admission** ***Abdominal*** ***Respiratory*** ***Soft tissue*** ***Urinary*** ***Endocarditis*** ***Neurological***	23, 35.7% 39, 55.7% 3, 4.3% 0, 0% 2, 2.9% 1, 1.4%	42, 60% 22, 31.4% 3, 4.3% 2, 2.9% 0, 0% 1, 1.4%	**0.023**		
**Patient category** ***Surgical*** ***Medical***	27, 38.6% 43, 61.4%	45, 64.3% 25, 35.7%	**0.002**	3.014 (1.217–7.466)	**0.017**
**Comorbidities** ***Cardiac*** ***Respiratory*** ***Neurologic*** ***Endocrinology*** ***Gastrointestinal system*** ***Malignancy***	45, 64.3% 33, 47.1% 20, 28.6% 31, 44.3% 15, 21.4% 28, 40%	43, 61.4% 17, 24.3% 17, 24.3% 24, 34.3% 24, 34.3% 31, 44.3%	0.726 **0.005** 0.565 0.226 0.09 0.608	3.001 (1.139–7.907)	**0.026**
**CCI**	6.0 (3.0–8.0)	5.0 (3.0–7.0)	0.379		
**APACHE II score (on the first day of admission)**	18.0 (12.8–23.3)	12.0 (10.0–18.3)	**0.007**	0.973 (0.911–1.039)	0.416
**GCS**	10.0 (8.0–11.3)	13.0 (10.0–13.0)	**<0.001**	0.737 (0.595–0.914)	**0.005**
**The initial SOFA score**	8.0 (5.0–10.0)	7.0 (5.0–8.0)	**0.012**	0.951 (0.780–1.159)	0.616
**ICU length of stay**	9.0 (5.0–19.0)	7.0 (4.0–15)	**0.036**		
**MV**	69, 98.6%	52, 74.3%	**<0.001**	6.321 (0.652–61.257)	0.112
**MV duration**	7.5 (3.0–16.3)	1.0 (0–3.0)	**<0.001**		
**Vasopressor support**	69, 98.6%	60, 85.7%	**0.005**	9.658 (0.497–187.815)	0.134
**Vasopressor support duration**	7.0 (3.8–16)	2.0 (1–4)	**<0.001**		
**AKI**	48, 68.6%	29, 41.4%	**0.001**	2.249 (0.791–6.399)	0.129
**RRT**	19, 27.1%	5, 7.1%	**0.002**	3.268 (0.871–12.260)	0.079
**Laboratory data**					
**WBC count (10^3^/µL)**	11.9 (8.1–15.7)	10.8 (7.1–17.3)	0.672		
**Neutrophil count (10^3^/µL)**	10.7 (6.1–14.3)	9.4 (5.6–15.1)	0.647		
**Lymphocyte count (10^3^/µL)**	0.8 (0.4–1.3)	0.8 (0.5–1.0)	0.487		
**NLR**	11.9 (5.3–22.6)	12.8 (6.9–22.8)	0.523		
**PLR**	258.2 (109.3–563.8)	358.9 (214.9–534.5)	0.111		
**CRP (mg/L)**	171.9 (64.1–286.5)	189.5 (80.0–288.7)	0.591		
**PCT (ng/mL)**	2.48 (0.54–14.8)	3.28 (0.60–18.3)	0.571		
**Creatinine (mg/dL)**	1.25 (0.74–2.22)	1.13 (0.7–1.7)	0.305		
**Albumin (g/dL)**	2.71 (2.43–3.15)	2.85 (2.53–3.25)	0.548		
**Lactate (mmol/L)**	1.9 (1.5–3.8)	1.85 (1.2–3.4)	0.510		
**NT-proBNP (µg/L)**	7566 (1885–18,027)	2617 (651–8204)	**0.004**	1 (1–1)	0.148
**D-dimer (µg/L)**	2316 (1021–4430)	1248 (944–4932)	0.270		
				*p* < 0.001,Nagelkerke R Square: 0.487	

BMI, body mass index; CCI, Charlson Comorbidity Index; APACHE, Acute Physiologic and Chronic Health Evaluation; GCS, Glasgow Coma Scale; SOFA, Sequential Organ Failure Assessment; ICU, intensive care unit; MV, mechanical ventilation; AKI, acute kidney injury; RRT, renal replacement therapy; WBC, white blood cell count; NLR, neutrophil–lymphocyte ratio; PLR, platelet–lymphocyte ratio; CRP, C-reactive protein; PCT, procalcitonin; NT-proBNP, N-terminal pro-B-type natriuretic peptide.

## Data Availability

The clinical data of this trial are available upon reasonable request to the corresponding author.

## Abbreviations

ICU, Intensive Care Unit; AKI, Acute Kidney Injury; S-AKI, Sepsis-associated Acute Kidney; Injury; RRT, Renal Replacement Therapy; SDC1, Syndecan-1; NGAL, Neutrophil Gelatinase-associated Lipocalin; PENK, Proenkephalin; PSPN, Presepsin; STROBE, Strengthening the Reporting of Observational Studies in Epidemiology; SOFA, Sequential Organ Failure Assessment; APACHE, Acute Physiology and Chronic Health Evaluation; KDIGO, Kidney Disease: Improving Global Outcomes; BMI, Body Mass Index; CCI, Charlson Comorbidity Index; GCS, Glasgow Coma Scale; WBC, White Blood Cell Count; CRP, C-Reactive Protein, PCT, Procalcitonin; NT-proBNP, N-Terminal Pro-B-Type Natriuretic Peptide; MV, Mechanical Ventilation; IQR, Interquartile Range; OR, Odds Ratio; ROC, Receiver Operating Characteristic; AUC, Area Under Curve; MV, Mechanical Ventilation; NLR, Neutrophil-to-Lymphocyte ratio; PLR, Platelet-to-Lymphocyte ratio.
